# Prognostic value of the preoperative C-reactive protein-to-lymphocyte ratio in patients with non-metastatic clear cell renal cell carcinoma

**DOI:** 10.1371/journal.pone.0362126

**Published:** 2026-10-30

**Authors:** Minchao Qin, Zhongwen Feng, Chao Zhang, Wei Song, Kai Wang, Zhen Chen

**Affiliations:** 1 Department of Urology, Jincheng People’s Hospital, Jincheng, Shanxi Province, China; 2 Department of Urology, Sir Run Run Hospital Nanjing Medical University, Nanjing, Jiangsu Province, China; 3 Department of Urology, The Third Affiliated Hospital of Soochow University, Changzhou, Jiangsu Province, China; Guangdong Nephrotic Drug Engineering Technology Research Center, Institute of Consun Co. for Chinese Medicine in Kidney Diseases, CHINA

## Abstract

**Background:**

Systemic inflammation is associated with the prognosis of clear cell renal cell carcinoma (ccRCC). The C-reactive protein-to-lymphocyte ratio (CLR) combines systemic inflammation and host immune status. However, its prognostic value in non-metastatic ccRCC remains unclear. This study evaluated the association between preoperative CLR and survival outcomes in patients with non-metastatic ccRCC.

**Methods:**

We retrospectively included 414 patients with non-metastatic ccRCC who underwent partial or radical nephrectomy. The primary Cox analyses treated ln(CLR) as a continuous variable and assessed overall survival (OS) and the cause-specific hazard of ccRCC-related death. Sensitivity analyses included expanded adjustment and time-varying effects. Cut-off-based analyses were exploratory. Time-dependent receiver operating characteristic curves were used to compare CLR with other inflammatory markers. Incremental discrimination and calibration were assessed using bootstrap internal validation.

**Results:**

After adjustment for age, Fuhrman grade, and T stage, ln(CLR) remained independently associated with OS (HR = 1.589, 95% CI 1.257–2.009; *P* < 0.001) and ccRCC-related death (HR = 1.612, 95% CI 1.266–2.052; *P* < 0.001). The associations persisted in sensitivity analyses addressing additional pathological factors and time-varying effects. The 3- and 5-year AUCs of CLR for OS were 0.810 and 0.776, respectively. No statistically significant AUC differences were detected between CLR and CRP, NLR, or CAR. Adding CLR increased the apparent C-index from 0.843 to 0.885. After bootstrap optimism correction, the C-indices were 0.832 and 0.873, respectively (ΔC-index = 0.041). Calibration was assessed at 3 and 5 years.

**Conclusions:**

Preoperative CLR was independently associated with OS and the cause-specific hazard of ccRCC-related death in this single-center cohort. Adding CLR improved discrimination after internal validation, but its superiority over other inflammatory markers and clinical utility remain unestablished. Independent multicenter validation is needed.

## Introduction

Renal cell carcinoma (RCC) is the most common form of kidney cancer, with clear cell renal cell carcinoma (ccRCC) accounting for approximately 70% of cases [1]. Surgery remains the main treatment for patients with non-metastatic RCC [1]. However, disease recurrence and cancer-related death may still occur after surgery [2]. Tumor stage and nuclear grade are well-established prognostic factors [3]. However, current clinicopathological factors do not fully account for differences in outcomes among patients with localized ccRCC [3]. Additional markers may therefore help improve postoperative risk assessment.

Systemic inflammation is closely associated with cancer progression and prognosis [4,5]. Several blood-based inflammatory markers have been investigated in patients with RCC [6]. C-reactive protein (CRP) is a widely used marker of systemic inflammation, and elevated CRP levels have been associated with poor outcomes in RCC [6,7]. Lymphocytes play an important role in antitumor immune responses, and a reduced lymphocyte count may reflect impaired host immunity [8]. Several indices derived from routine blood parameters have also been evaluated for prognostic assessment in RCC. Among these, the neutrophil-to-lymphocyte ratio (NLR) and C-reactive protein-to-albumin ratio (CAR) have shown prognostic value in previous studies [9,10].

The C-reactive protein-to-lymphocyte ratio (CLR) combines CRP and lymphocyte count and may reflect both systemic inflammation and host immune status [11,12]. A high CLR can result from an increased CRP level, a decreased lymphocyte count, or both. Previous studies have reported an association between CLR and prognosis in several types of cancer [11–14]. However, its prognostic role in ccRCC remains unclear. Whether CLR can provide additional prognostic information beyond conventional clinicopathological factors also requires further study.

This study evaluated the prognostic value of preoperative CLR in patients with non-metastatic ccRCC undergoing nephrectomy. We examined the associations of CLR with overall survival (OS) and cancer-specific survival (CSS). We also compared its prognostic performance with CRP and other inflammatory indices, including CNR, CMR, NLR, and CAR. Finally, we assessed whether adding CLR to conventional clinicopathological factors improved prognostic performance.

## Materials and methods

### Study population

This retrospective study included patients with histologically confirmed ccRCC who underwent partial or radical nephrectomy at the Third Affiliated Hospital of Soochow University between 2003 and 2012. The study was conducted in accordance with the 2013 revision of the Declaration of Helsinki and was approved by the hospital’s Medical Ethics Committee (Approval No. 2013−14). The approved protocol covered the retrospective analysis of clinicopathological and follow-up data from this patient population, and the Ethics Committee waived the requirement for written informed consent. Medical records were accessed for research purposes in 2014. The investigators had access to identifiable patient information during data collection, but all data were de-identified before statistical analysis, and no directly identifiable information was included in the analytical dataset. Eligible patients had pathologically confirmed ccRCC, underwent surgical treatment, and had available clinicopathological and preoperative laboratory data. Patients with distant metastasis at diagnosis, other malignancies, a history of preoperative neoadjuvant therapy, active infections, chronic inflammatory diseases, hematological disorders, or autoimmune diseases were excluded. Patients with incomplete clinical data or insufficient follow-up information were also excluded. Finally, 414 patients were included in the analysis.

### Data collection and definition of inflammatory indices

The collected clinicopathological data included age at surgery, sex, tumor size, Fuhrman grade, pathological T stage, pathological N stage, lymphvascular invasion (LVI), and tumor necrosis. Tumor stage was determined according to the 2010 American Joint Committee on Cancer (AJCC) TNM staging system. Tumor size was defined as the maximum diameter of the primary tumor. Tumor necrosis was defined as microscopic coagulative necrosis. LVI was defined as the presence of tumor cells within endothelial-lined vascular or lymphatic spaces without an identifiable muscular wall. Preoperative laboratory data was obtained from routine blood tests performed within one week before surgery. The variables included CRP(mg/L), neutrophil count (×10^9/L), lymphocyte count (×10^9/L), monocyte count (×10^9/L), and serum albumin (g/L). All laboratory measurements were performed in the hospital laboratory according to routine standardized procedures. The inflammatory indices were calculated using preoperative laboratory values as follows: CLR, CRP/absolute lymphocyte count; CNR, CRP/absolute neutrophil count; CMR, CRP/absolute monocyte count; NLR, absolute neutrophil count/absolute lymphocyte count; and CAR, CRP/serum albumin.

### Determination of the CLR cut-off

The optimal CLR cut-off for OS was determined using maximally selected log-rank statistics, requiring each group to contain at least 10% of patients. To account for multiple testing across candidate cut-offs, a search-adjusted P value was estimated using 9,999 conditional Monte Carlo simulations. A cut-off value of 4.298, rounded to 4.3 for reporting, was identified, and patients were divided into low-CLR (≤4.3) and high-CLR (>4.3) groups. Cut-off stability was assessed using 1,000 bootstrap samples drawn with replacement from the original cohort. The entire cut-off selection procedure was repeated in each sample using the same selection criteria. The distribution of bootstrap cut-offs was summarized using the median, IQR, and 2.5th–97.5th percentiles.

### Follow-up and outcomes

Follow-up data were collected from outpatient records, telephone follow-up, and electronic medical records. OS was calculated from the date of surgery to death from any cause or the last follow-up.CSS was calculated from the date of surgery to death due to ccRCC. In the CSS analysis, deaths from other causes were censored at the date of death, and patients alive at the last follow-up were censored at that time. The CSS Cox models estimated the cause-specific hazard of ccRCC-related death rather than its absolute cumulative incidence. Median follow-up duration and its interquartile range were estimated using the reverse Kaplan–Meier method. Numbers at risk were included in the Kaplan–Meier plots.

### Statistical analysis

Continuous variables are reported as medians and interquartile ranges (IQRs), while categorical variables are presented as numbers and percentages. Differences between the CLR groups were assessed using the Mann–Whitney U test for continuous variables. The chi-square test or Fisher’s exact test was used for categorical variables, as appropriate. Kaplan–Meier curves were used to estimate OS and CSS, and differences between groups were assessed using the log-rank test. The conventional log-rank P values shown in the Kaplan–Meier plots were unadjusted for cut-off selection; the search-adjusted P value for OS was reported separately. Restricted cubic spline analysis was performed to examine the association between ln (CLR) and mortality risk. Four knots were used to allow flexibility while limiting model complexity, located at ln(CLR) values of −0.351, 0.352, 0.744, and 2.941, corresponding to CLR values of 0.704, 1.422, 2.104, and 18.937. The reference value was the median ln(CLR) of 0.535, corresponding to a CLR of 1.708. The model was adjusted for age, Fuhrman grade (1–2 vs. 3–4), and T stage (T1a, T1b–T2, and T3–4). The overall association and departure from linearity were assessed using Wald tests, with the nonlinear spline terms tested jointly. Univariable and multivariable Cox regression analyses were performed for OS and CSS. The CSS models estimated cause-specific hazard ratios for ccRCC-related death, with deaths from other causes treated as censored observations. These analyses assessed prognostic associations rather than the absolute cumulative probability of cancer-related death. The multivariable models included age, Fuhrman grade, T stage, and ln (CLR). Hazard ratios (HRs) and 95% confidence intervals (CIs) were calculated. The main Cox analyses treated ln(CLR) as a continuous variable, whereas cut-off-based survival and subgroup analyses were considered exploratory. The proportional-hazards assumption was assessed using scaled Schoenfeld residual tests with a Kaplan–Meier transformation of time. Term-specific and global tests were performed for the primary and expanded Cox models. Because departures from the proportional-hazards assumption were detected, supplementary Cox models included interactions of ln(CLR), Fuhrman grade, and both T-stage indicators with log(t/36), where t denotes months since surgery. The 36-month value was used only for centering, not as a temporal cut-off. Likelihood ratio tests compared the model containing all four-time interactions with the conventional model. The ln(CLR) time interaction was also tested while retaining the time interactions for Fuhrman grade and T stage. Time-specific HRs and pointwise 95% CIs for ln(CLR) were estimated at 12, 36, and 60 months. Time-dependent receiver operating characteristic (ROC) curves were used to assess the prognostic performance of CLR, CRP, CNR, CMR, NLR, and CAR in 3 and 5 years. The AUCs of CLR were compared with those of the other inflammatory markers. A clinical model including age, Fuhrman grade, and T stage was used as the reference model. The incremental prognostic value of each inflammatory marker was evaluated using the concordance index (C-index), Akaike information criterion (AIC), and likelihood ratio test (LRT). Subgroup analyses were performed according to sex, age, Fuhrman grade, T stage, N stage, lymphvascular invasion, tumor size, and tumor necrosis. Interaction terms were used to assess differences in the association between CLR and OS across subgroups. Pairwise AUC comparisons and subgroup interaction analyses were exploratory. Their P values were not adjusted for multiple testing and should be interpreted cautiously. Sensitivity analyses additionally adjusted for tumor size, N stage, lymphovascular invasion, and tumor necrosis. The original and expanded models were fitted in the same complete-case sample. Given the limited number of events, ridge-penalized Cox models were also fitted using the expanded covariate set. Continuous predictors were standardized internally, and the penalty was selected by five-fold cross-validation stratified by event status, using partial-likelihood deviance and the one-standard-error rule. Penalized coefficients were reported on their original scales. All statistical analyses were performed using R software (version 4.6.0; R Foundation for Statistical Computing, Vienna, Austria). All tests were two-sided, and a P value <0.05 was considered statistically significant.

## Results

### Patient characteristics and distribution of CLR

A total of 414 patients with non-metastatic ccRCC were included in the analysis. Using the cohort-derived CLR cut-off of 4.3, 351 patients were classified into the low-CLR group and 63 into the high-CLR group (Table 1). The median age of the overall cohort was 57 years, and 62.1% of the patients were male. Patients with high CLR had larger tumors than those with low CLR [6.00 (3.50–8.00) vs. 4.00 (3.00–6.00) cm, *P <* 0.001]. High CLR was also associated with higher Fuhrman grade, more advanced T stage, lymphovascular invasion, and tumor necrosis (all *P <* 0.001). No significant differences were observed in sex, age, or lymph node status. As expected, the high-CLR group had higher CRP levels and lower lymphocyte counts. Higher neutrophil and monocyte counts, lower albumin levels, higher NLR, and higher CAR were also observed in this group (all *P <* 0.001). The distribution of CLR was right-skewed, and maximally selected rank statistics identified the optimal cut-off value shown in Fig 1A. Maximally selected log-rank statistics identified a CLR cut-off of 4.298, reported as 4.3, with a search-adjusted P value <0.001. Across 1,000 bootstrap samples, the median selected cut-off was 4.565 (IQR, 4.298–5.962), with a 2.5th–97.5th percentile range of 2.453–7.083. The full sample cut-off was the most frequently selected value, occurring in 203 samples (20.3%) (S1 Fig).

**Table 1:** Baseline clinicopathological characteristics of patients according to preoperative CLR.

Characteristic	Overall (N = 414)	Low CLR (n = 351)	High CLR (n = 63)	*P* value
Sex				0.130
Male	257 (62.1%)	212 (60.4%)	45 (71.4%)	
Female	157 (37.9%)	139 (39.6%)	18 (28.6%)	
Age, years	57.00 (48.00–64.00)	57.00 (48.00–64.00)	60.00 (51.00–68.00)	0.110
Tumor size, cm	4.00 (3.00–6.00)	4.00 (3.00–6.00)	6.00 (3.50–8.00)	<0.001
Fuhrman grade				<0.001
1–2	294 (71.0%)	261 (74.4%)	33 (52.4%)	
3–4	120 (29.0%)	90 (25.6%)	30 (47.6%)	
T stage				<0.001
T1a	201 (48.6%)	180 (51.3%)	21 (33.3%)	
T1b–T2	181 (43.7%)	159 (45.3%)	22 (34.9%)	
T3–4	32 (7.7%)	12 (3.4%)	20 (31.7%)	
Lymph node status				0.078
N0	404 (97.6%)	345 (98.3%)	59 (93.7%)	
N1	10 (2.4%)	6 (1.7%)	4 (6.3%)	
Lymphovascular invasion				<0.001
Absent	393 (94.9%)	343 (97.7%)	50 (79.4%)	
Present	21 (5.1%)	8 (2.3%)	13 (20.6%)	
Tumor necrosis				<0.001
Absent	375 (90.6%)	331 (94.3%)	44 (69.8%)	
Present	39 (9.4%)	20 (5.7%)	19 (30.2%)	
C-reactive protein, mg/L	3.00 (2.10–4.20)	2.60 (2.00–3.70)	13.80 (9.60–30.20)	<0.001
Lymphocyte count, × 10^9^/L	1.76 (1.34–2.10)	1.81 (1.44–2.14)	1.36 (1.04–1.70)	<0.001
Neutrophil count, × 10^9^/L	3.69 (2.93–4.60)	3.55 (2.85–4.39)	5.03 (3.60–6.82)	<0.001
Monocyte count, × 10^9^/L	0.45 (0.36–0.57)	0.44 (0.35–0.54)	0.59 (0.38–0.73)	<0.001
Albumin, g/L	40.90 (38.30–43.00)	41.30 (38.70–43.40)	37.20 (33.30–41.00)	<0.001
Neutrophil-to-lymphocyte ratio	2.15 (1.57–2.86)	2.00 (1.48–2.58)	3.64 (2.50–5.30)	<0.001
CRP-to-albumin ratio	0.07 (0.05–0.11)	0.06 (0.05–0.09)	0.37 (0.25–0.89)	<0.001

Continuous variables are presented as median (interquartile range), and categorical variables as number (percentage). Patients were classified into low-CLR (≤4.3) and high-CLR (>4.3) groups. **Abbreviations:** CLR, C-reactive protein-to-lymphocyte ratio; CRP, C-reactive protein.

**Fig 1:**
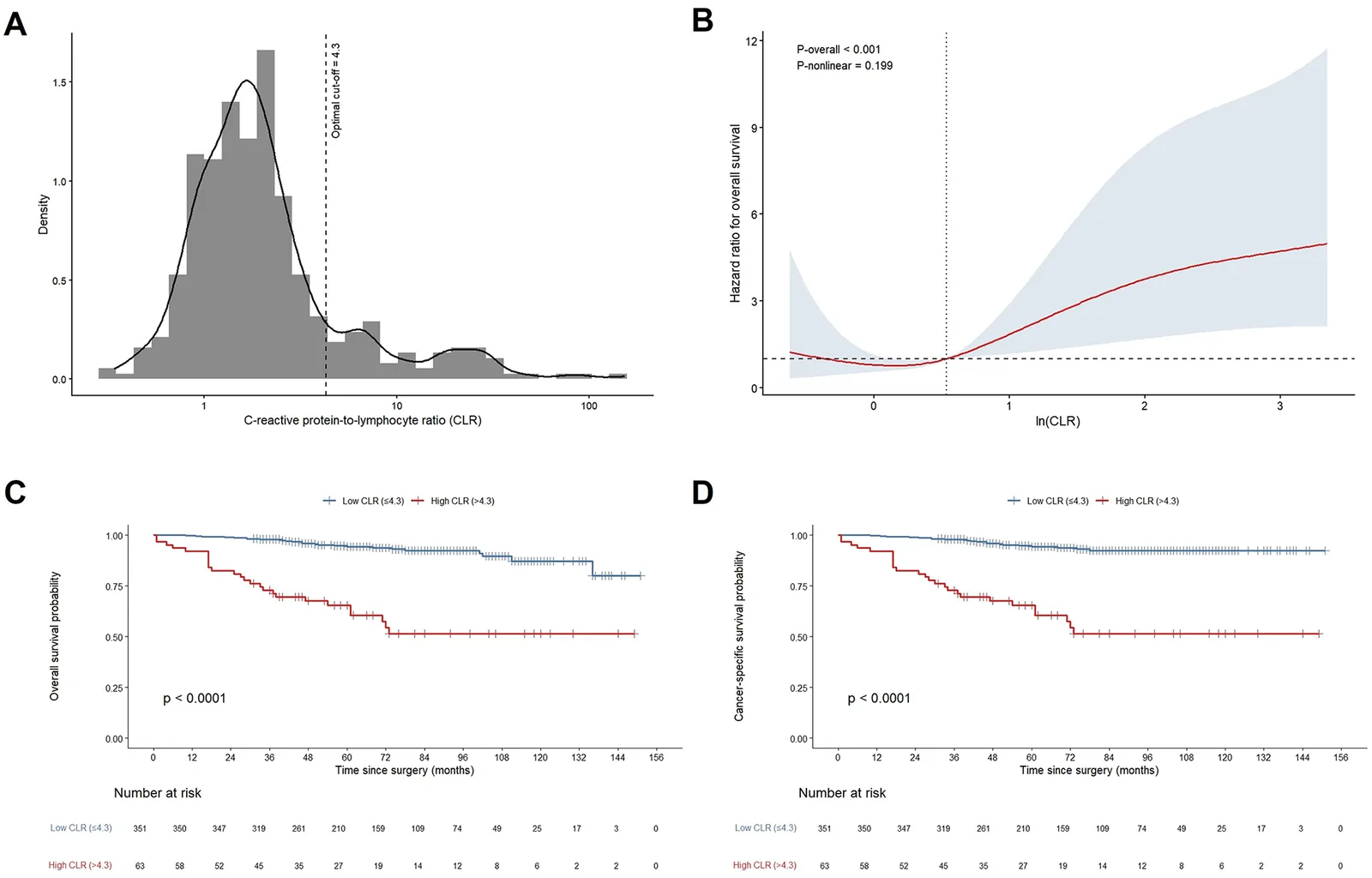
Distribution of CLR and survival analyses according to preoperative CLR in patients with ccRCC. (A) Distribution of preoperative CLR. The dashed vertical line indicates the optimal cut-off value of 4.3. (B) Restricted cubic spline analysis of the association between ln (CLR) and overall survival. The solid line represents the estimated hazard ratio, and the shaded area represents the 95% confidence interval. (C) Kaplan–Meier curves for overall survival according to CLR group. (D) Kaplan–Meier curves for cancer-specific survival according to CLR group. Differences between groups were assessed using the log-rank test. **Abbreviations**: CLR, C-reactive protein-to-lymphocyte ratio; ccRCC, clear cell renal cell carcinoma.

### Association between CLR and survival outcomes

The median follow-up duration, estimated using the reverse Kaplan–Meier method, was 70 months (IQR, 49–95 months). Restricted cubic spline analysis, adjusted for age, Fuhrman grade, and T stage, showed a significant association between ln(CLR) and all-cause mortality (*P* for overall association <0.001), with no evidence of departure from linearity (*P* for nonlinearity = 0.199; Fig 1B). The estimated hazard of death generally increased with increasing ln(CLR). Kaplan–Meier analysis showed that patients with high CLR had worse OS and CSS than those with low CLR (both log-rank *P* < 0.0001; Fig 1C and 1D).

### Independent prognostic value of CLR

In univariable Cox analysis for OS, higher ln(CLR) was associated with an increased risk of death (HR 2.154, 95% CI 1.774–2.614; *P <* 0.001) in Table 2. After adjustment for age, Fuhrman grade, and T stage, ln(CLR) remained independently associated with OS (HR 1.589, 95% CI 1.257–2.009; *P <* 0.001). Advanced T stage also remained an independent predictor of poor OS. Similar results were observed for CSS. Higher ln(CLR) was associated with cancer-specific mortality in univariable analysis (HR 2.212, 95% CI 1.820–2.690; *P <* 0.001). This association remained significant in the multivariable model (HR 1.612, 95% CI 1.266–2.052; *P <* 0.001). T stage was also independently associated with CSS.

**Table 2:** Univariable and multivariable Cox regression analyses of overall survival and cancer-specific survival in patients with ccRCC.

Variable	Univariable HR (95% CI)	P value	Multivariable HR (95% CI)	P value
**Panel A. Overall survival**				
Sex		0.156		
Female vs. Male	0.639 (0.345–1.190)			
Age, per year	1.031 (1.005–1.058)	0.019	1.019 (0.991–1.048)	0.178
Tumor size, per cm	1.217 (1.125–1.316)	<0.001		
Fuhrman grade		<0.001		0.596
3–4 vs. 1–2	3.154 (1.802–5.520)		1.190 (0.626–2.262)	
T stage		<0.001		<0.001
T1a	Reference		Reference	
T1b–T2	4.308 (1.754–10.584)	0.001	3.655 (1.467–9.104)	0.005
T3–4	41.511 (16.622–103.672)	<0.001	18.709 (6.532–53.588)	<0.001
N stage		<0.001		
N1 vs. N0	8.350 (3.530–19.700)			
Lymphovascular invasion		<0.001		
Present vs. Absent	8.263 (4.199–16.260)			
Tumor necrosis		<0.001		
Present vs. Absent	4.020 (2.190–7.390)			
ln (CLR)	2.154 (1.774–2.614)	<0.001	1.589 (1.257–2.009)	<0.001
**Panel B. Cancer-specific survival**				
**Variable**	**Univariable HR (95% CI)**	**P value**	**Multivariable HR (95% CI)**	**P value**
Sex		0.179		
Female vs. Male	0.644 (0.339–1.220)			
Age, per year	1.030 (1.003–1.057)	0.031	1.015 (0.987–1.045)	0.297
Tumor size, per cm	1.234 (1.138–1.337)	<0.001		
Fuhrman grade		<0.001		0.652
3–4 vs. 1–2	3.341 (1.865–5.986)		1.166 (0.598–2.276)	
T stage		<0.001		<0.001
T1a	Reference		Reference	
T1b–T2	5.921 (2.032–17.251)	0.001	5.102 (1.730–15.044)	0.003
T3–4	60.003 (20.507–175.570)	<0.001	26.923 (8.091–89.583)	<0.001
N stage		<0.001		
N1 vs. N0	8.350 (3.530–19.700)			
Lymphovascular invasion		<0.001		
Present vs. Absent	8.818 (4.452–17.470)			
Tumor necrosis		<0.001		
Present vs. Absent	4.820 (2.600–8.940)			
ln (CLR)	2.212 (1.820–2.690)	<0.001	1.612 (1.266–2.052)	<0.001

Multivariable Cox regression models included age, Fuhrman grade, T stage, and ln (CLR). Hazard ratios are presented with 95% confidence intervals. **Abbreviations**: CLR, C-reactive protein-to-lymphocyte ratio; ccRCC, clear cell renal cell carcinoma; OS, overall survival; CSS, cancer-specific survival; HR, hazard ratio; CI, confidence interval.

### Proportional-hazards assessment

The global Schoenfeld residual tests indicated departures from the proportional-hazards assumption in the primary OS and CSS models (*P* = 0.023 and *P* = 0.024, respectively). Term-specific tests identified departures for Fuhrman grade and T stage in both models. For ln(CLR), the test was significant in the OS model (*P* = 0.026) but not in the CSS model (*P* = 0.070). Similar findings were observed in the expanded models, with significant tests for Fuhrman grade and T stage in both outcomes and for ln(CLR) in OS (*P* = 0.027)(S2 Table). Adding time interactions for ln(CLR), Fuhrman grade, and T stage improved model fit for OS and CSS compared with the conventional models (likelihood ratio *P =* 0.004 and *P =* 0.012, respectively). With the pathological time interactions retained, the ln(CLR) time interaction was not statistically significant for either outcome (OS, *P =* 0.253; CSS, *P =* 0.331). The estimated HRs for ln(CLR) at 12, 36, and 60 months were 1.877, 1.572, and 1.448 for OS, and 1.876, 1.594, and 1.478 for CSS, respectively. All corresponding pointwise 95% CIs excluded 1 (S3 Table).

### Comparative prognostic performance of CLR and other inflammatory markers

During follow-up, 50 deaths occurred, of which 46 were cancer specific. All four non-cancer deaths occurred after 60 months. The estimated 3- and 5-year time-dependent AUCs for OS and CSS were identical in this analysis. Only the OS results are presented in Fig 2 and Table 3. CLR yielded AUCs of 0.810 (95% CI 0.690–0.930) at 3 years and 0.776 (95% CI 0.685–0.867) at 5 years. No statistically significant differences in AUC were detected between CLR and CRP, NLR, or CAR at either time point (all *P* > 0.05). In exploratory pairwise comparisons, CLR showed higher AUCs than CNR and CMR at both time points (all nominal P < 0.05). These P values were not adjusted for multiple testing (all *P <* 0.05).

**Table 3:** Time-dependent discriminatory performance of CLR and other inflammatory markers for overall survival.

Marker	3-year AUC (95% CI)	*P* vs CLR	5-year AUC (95% CI)	*P* vs CLR
CLR	0.810 (0.690–0.930)	Reference	0.776 (0.685–0.867)	Reference
CRP	0.811 (0.693–0.928)	0.968	0.756 (0.659–0.854)	0.335
CNR	0.742 (0.609–0.874)	0.032	0.672 (0.561–0.783)	<0.001
CMR	0.774 (0.650–0.897)	0.025	0.708 (0.608–0.809)	0.001
NLR	0.794 (0.721–0.867)	0.748	0.770 (0.697–0.843)	0.895
CAR	0.821 (0.707–0.936)	0.584	0.768 (0.675–0.861)	0.660

Time-dependent AUCs were estimated at 3 and 5 years. P values represent pairwise comparisons between CLR and each inflammatory marker. **Abbreviations**: AUC, area under the curve; CLR, C-reactive protein-to-lymphocyte ratio; CRP, C-reactive protein; CNR, C-reactive protein-to-neutrophil ratio; CMR, C-reactive protein-to-monocyte ratio; NLR, neutrophil-to-lymphocyte ratio; CAR, C-reactive protein-to-albumin ratio; OS, overall survival.

**Fig 2:**
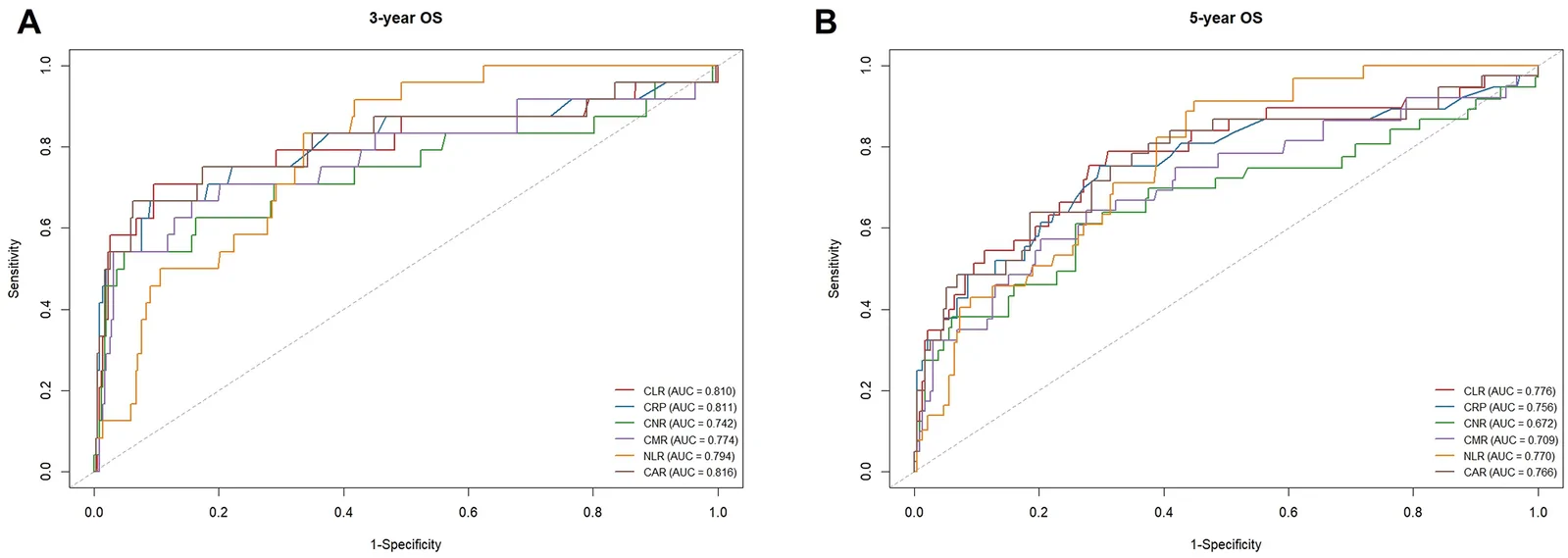
Time-dependent ROC curves of CLR and other inflammatory markers for overall survival. Time-dependent ROC curves comparing the discriminatory performance of CLR, CRP, CNR, CMR, NLR, and CAR for (A) 3-year and (B) 5-year overall survival. AUC values for each marker are shown in the figure. **Abbreviations**: ROC, receiver operating characteristic; AUC, area under the curve; CLR, C-reactive protein-to-lymphocyte ratio; CRP, C-reactive protein; CNR, C-reactive protein-to-neutrophil ratio; CMR, C-reactive protein-to-monocyte ratio; NLR, neutrophil-to-lymphocyte ratio; CAR, C-reactive protein-to-albumin ratio; OS, overall survival.

### Incremental prognostic value

The clinical model, including age, Fuhrman grade, and T stage, had an apparent C-index of 0.843 and an AIC of 488.7. Adding CLR increased the apparent C-index to 0.885 and reduced the AIC to 476.6 (ΔC-index=+0.042; ΔAIC=−12.1; LRT *P <* 0.001). CRP, NLR, and CAR also improved the apparent performance of the clinical model, whereas smaller changes were observed for CNR and CMR (Table 4).

**Table 4:** Incremental prognostic value of inflammatory markers beyond the clinical model for overall survival.

Model	C-index (95% CI)	ΔC-index	AIC	ΔAIC	LRT *P* value
Clinical model	0.843 (0.788–0.898)	Reference	488.7	Reference	—
+ CLR	0.885 (0.838–0.933)	+0.042	476.6	−12.1	<0.001
+ CRP	0.885 (0.837–0.933)	+0.042	474.4	−14.3	<0.001
+ CNR	0.866 (0.815–0.918)	+0.023	485.2	−3.5	0.019
+ CMR	0.867 (0.814–0.920)	+0.024	485.1	−3.6	0.018
+ NLR	0.879 (0.830–0.928)	+0.036	477.6	−11.1	<0.001
+ CAR	0.888 (0.841–0.934)	+0.045	472.3	−16.3	<0.001

The clinical model included age, Fuhrman grade, and T stage. ΔC-index and ΔAIC were calculated relative to the clinical model. Likelihood ratio tests compared each extended model with the clinical model. **Abbreviations:** CLR, C-reactive protein-to-lymphocyte ratio; CRP, C-reactive protein; CNR, C-reactive protein-to-neutrophil ratio; CMR, C-reactive protein-to-monocyte ratio; NLR, neutrophil-to-lymphocyte ratio; CAR, C-reactive protein-to-albumin ratio; AIC, Akaike information criterion.

### Internal validation of model performance

Of 1,000 paired bootstrap repetitions, 999 were successfully completed for discrimination assessment. The optimism-corrected C-indices were 0.832 for the clinical model and 0.873 after adding ln(CLR), corresponding to an increase of 0.041 compared with the apparent increase of 0.042 (S4 Table). For calibration, 997 of 1,000 repetitions were retained for each model and time point; three were excluded because of potentially infinite coefficients. The mean absolute calibration errors at 3 and 5 years were 0.0274 and 0.0138 for the clinical model, and 0.0245 and 0.0149 for the model including CLR. Adding CLR did not consistently reduce calibration error (S4 Table; S2 Fig).

### Subgroup analyses

Exploratory subgroup analyses yielded an unadjusted *P* value of 0.010 for the T-stage interaction. In patients with T1b–T2 disease, the estimated HR for high versus low CLR was 9.00 (95% CI, 3.95–20.51), with substantial uncertainty in the effect magnitude reflected by the wide confidence interval. The associations were not statistically significant in the T1a and T3–4 subgroups. The remaining seven interaction tests did not reach nominal significance (sex, age, Fuhrman grade, N stage, lymphovascular invasion, tumor size, and tumor necrosis; all unadjusted *P* for interaction>0.05; Fig 3). These findings should be interpreted cautiously given multiple testing and sparse events in some subgroups.

**Fig 3:**
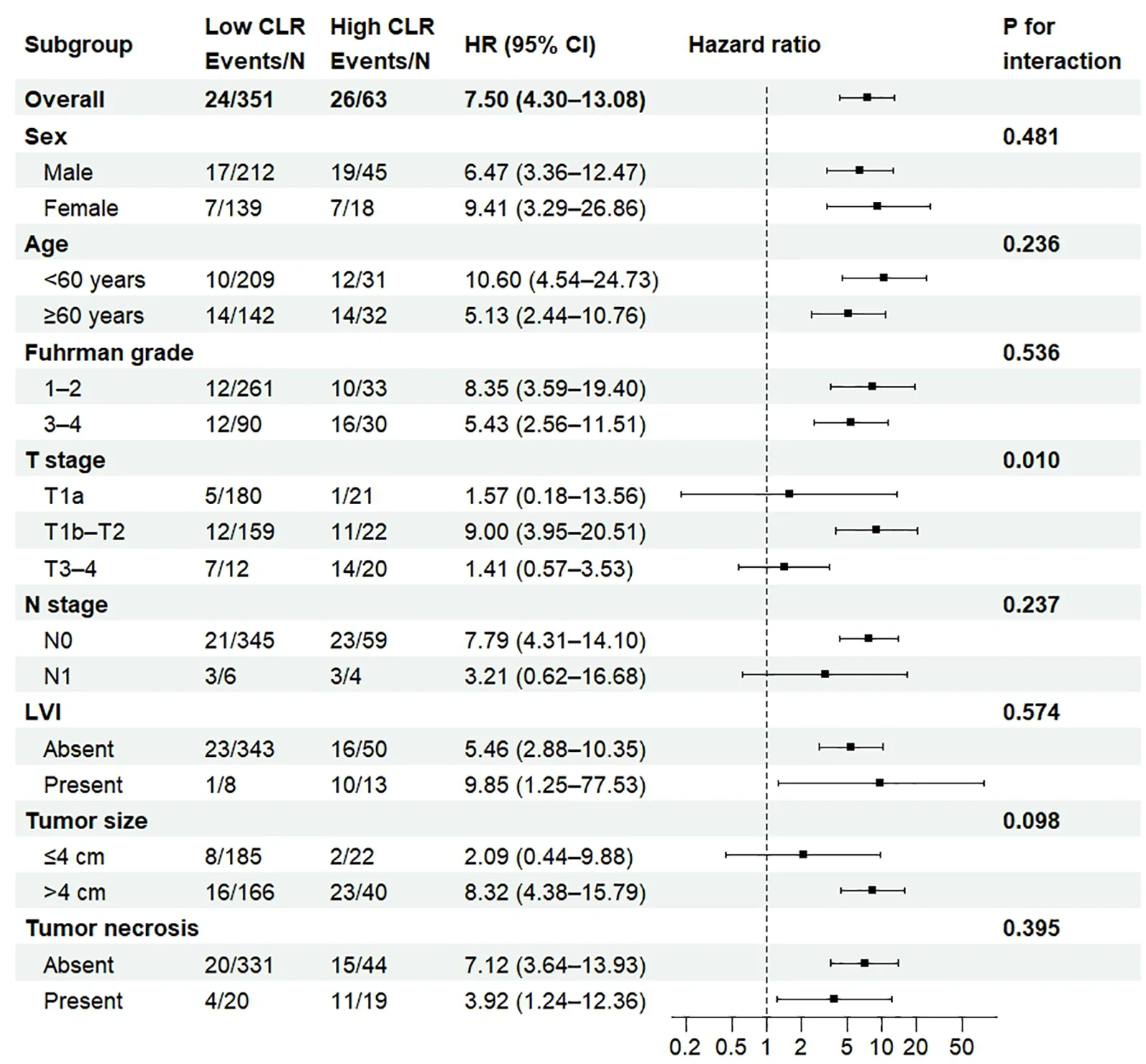
Subgroup analysis of the association between preoperative CLR and overall survival. Forest plot showing hazard ratios (HRs) and 95% confidence intervals (CIs) for high versus low CLR across prespecified subgroups. Patients were classified into low-CLR (≤4.3) and high-CLR (>4.3) groups. P values for interaction were calculated to assess differences in the association between CLR and overall survival across subgroups. **Abbreviations**: CLR, C-reactive protein-to-lymphocyte ratio; HR, hazard ratio; CI, confidence interval; LVI, lymphvascular invasion.

### Sensitivity analyses

The sensitivity analyses included 413 patients with complete covariate data, comprising 49 OS events and 45 CSS events. One patient was excluded because of missing tumor size. In this sample, the original model yielded HRs for ln(CLR) of 1.573 (95% CI, 1.242–1.993) for OS and 1.596 (95% CI, 1.251–2.036) for CSS. After additional adjustment for tumor size, N stage, lymphovascular invasion, and tumor necrosis, the corresponding HRs were 1.623 (95% CI, 1.240–2.122) and 1.638 (95% CI, 1.245–2.154), respectively (both *P <* 0.001). In ridge-penalized Cox models using the same expanded covariate set, the HRs for ln(CLR) were attenuated but remained above 1. Using the one-standard-error rule to select the penalty, the HRs were 1.121 for OS and 1.112 for CSS. Using the penalty that minimized cross-validated deviance, the corresponding HRs were 1.250 and 1.305 (S1 Table).

## Discussion

In this study, we evaluated the prognostic value of preoperative CLR in patients with non-metastatic ccRCC. High CLR was associated with several adverse pathological features, including larger tumor size, higher Fuhrman grade, advanced T stage, lymphovascular invasion, and tumor necrosis. Patients with high CLR also had worse OS and CSS. After adjustment for age, Fuhrman grade, and T stage, ln(CLR) remained independently associated with both outcomes. In addition, CLR showed discriminatory performance comparable to CRP, NLR, and CAR. Adding CLR to the clinical model improved model performance within this cohort. Supplementary models allowing time-varying effects supported the association between CLR and survival, although the limited number of events and the assumed log-time form warrant caution. These findings support a prognostic association between CLR and survival, but do not establish its clinical utility. Whether CLR can improve treatment or follow-up decisions requires prospective multicenter evaluation with independent validation of both the cut-off and prognostic performance.

CRP is a major acute-phase protein and a widely used marker of systemic inflammation [15,16]. It is mainly synthesized by hepatocytes in response to pro-inflammatory cytokines, particularly interleukin-6 (IL-6) [15]. During inflammatory responses, circulating CRP levels can increase rapidly and substantially. CRP also participates in innate immunity through complement activation, phagocytosis, and interactions with inflammatory cells [15]. Therefore, circulating CRP provides a simple measure of the systemic inflammatory state. In contrast, lymphocytes are key components of host immune defense and play an important role in antitumor immune surveillance [17]. This is particularly relevant to ccRCC, which is characterized by a highly infiltrated and heterogeneous immune microenvironment [18,19]. Cytotoxic T lymphocytes can recognize tumor-associated antigens and directly eliminate tumor cells [20]. While CD4 + T cells regulate and support antitumor immune responses [21]. Natural killer cells can recognize and destroy abnormal cells and are important effector lymphocytes of the innate immune system [22]. A reduced peripheral lymphocyte count may therefore indicate impaired cellular immunity and a weakened capacity for antitumor immune surveillance [17,21,22].

CLR incorporates these two components into a single index. Therefore, a high CLR may represent a state of increased systemic inflammation together with reduced immune activity. This may partly explain its association with adverse pathological features and poor survival in our cohort. The prognostic value of CLR has been investigated in several malignancies [11,12,23]. Previous studies in patients with non-small cell lung cancer showed that elevated CLR was independently associated with unfavorable survival outcomes after adjustment for conventional clinicopathological factors [11,12]. More recently, a meta-analysis including 22 studies and 7,220 patients provided broader evidence for the prognostic relevance of CLR across different cancer types [23]. Elevated CLR was significantly associated with worse OS, PFS, and DFS, suggesting that CLR may capture prognostically relevant information across different tumor settings [23]. Our findings extend these observations to non-metastatic ccRCC. The restricted cubic spline analysis showed that mortality risk increased with increasing ln (CLR), with no evidence of a significant nonlinear association. Moreover, ln (CLR) remained associated with both OS and CSS after adjustment for conventional clinicopathological factors. These results suggest that the prognostic information provided by CLR is not limited to a single data-derived cut-off.

In recent years, increasing attention has been paid to composite indices that integrate inflammatory and immune-related components for risk stratification. Several circulating markers, including CRP, NLR, PLR, LMR, SII, SIRI, and CAR, have been investigated as potential prognostic markers in patients with RCC [7,10,24–26]. These markers provide indirect information on systemic inflammation and host immune status. An important part of this study was the comparison of CLR with other inflammatory markers. CLR had 3- and 5-year AUCs of 0.810 and 0.776 for OS. No statistically significant differences in AUC were detected between CLR and CRP, NLR, or CAR; this does not establish equivalence. In contrast, CLR showed higher AUCs than CNR and CMR at both time points. Among the examined extended models, CAR had the highest apparent C-index (0.888) and the lowest AIC (472.3). CRP yielded essentially the same apparent C-index as CLR (both 0.885) and a lower AIC (474.4 vs. 476.6). These findings do not demonstrate superior prognostic performance of CLR over CRP, NLR, or CAR. Although CLR was associated with survival and improved discrimination beyond the clinical model, its added value over existing inflammatory markers remains uncertain.

Several limitations should be considered. First, this was a retrospective study from a single center, which may have introduced selection bias and limited generalizability. Residual confounding may remain despite multivariable adjustment and sensitivity analyses. Second, CLR was assessed from a single preoperative blood sample. Changes in CLR during follow-up were not evaluated. Third, the optimal cut-off was derived from the present cohort and may not be directly applicable to other populations. Although the cut-off search was accounted for statistically, bootstrap resampling showed variability in the selected threshold. The cut-off-based analyses should therefore be considered exploratory. Continuous ln(CLR) was used in the primary Cox analyses. Fourth, the number of outcome events was relatively limited, particularly for CSS. Unequal CLR group sizes (351 versus 63) and sparse events in some subgroups limited the precision and stability of the estimates. Multiple exploratory AUC comparisons and subgroup interaction tests were performed without multiplicity adjustment. The T-stage interaction and large subgroup HRs should therefore be interpreted cautiously. Departures from the proportional-hazards assumption were detected, including ln(CLR) in the OS model. Although time-varying sensitivity analyses were performed, the limited event count and assumed form of the time interactions constrain their interpretation. Conventional Cox HRs should not be interpreted as effects that necessarily remain constant throughout follow-up. The CSS models estimated cause-specific hazards rather than the absolute cumulative incidence of cancer-related death. Finally, adding CLR improved discrimination after bootstrap optimism correction, but internal validation does not establish transportability or clinical utility. This study did not include an independent external validation cohort. Prospective multicenter studies are needed to validate the cut-off and prognostic performance and determine the clinical utility of CLR.

## Conclusion

Preoperative CLR was independently associated with OS and CSS in patients with non-metastatic ccRCC in this single-center retrospective cohort. Adding CLR improved discrimination beyond the clinical model after bootstrap correction, but superiority over CRP, NLR, or CAR was not demonstrated. These findings support further investigation of CLR as a supplementary prognostic marker but do not establish its clinical utility. Prospective multicenter studies with independent validation of both the cut-off and prognostic performance are needed before clinical application.

## Supporting information

S1 Fig Bootstrap distribution of CLR cut-offs. Cut-off selection was repeated in 1,000 bootstrap samples using maximally selected log-rank statistics. The solid red line indicates the full-sample cut-off (4.298), and the dashed blue line indicates the reported cut-off (4.3).
(TIF)

S2 Fig Bootstrap-corrected calibration curves for overall survival. Panels show the clinical model at 3 years (A), the clinical model with CLR at 3 years (B), the clinical model at 5 years (C), and the clinical model with CLR at 5 years (D). The clinical model included age, Fuhrman grade, and T stage; CLR was entered as ln(CLR). Solid red lines show optimism-corrected calibration, dashed blue lines show apparent calibration, and dotted gray lines indicate perfect calibration. Each curve was based on 997 valid bootstrap repetitions and evaluated over the 5th–95th percentiles of the model’s predicted survival probabilities.
(TIF)

S1 Table Sensitivity analyses of the association between ln(CLR) and overall and cancer-specific survival. All models were fitted in the same complete-case sample of 413 patients, with 49 OS events and 45 CSS events. HRs represent a one-unit increase in ln(CLR). The primary Cox model included age, Fuhrman grade (1–2 vs. 3–4), T stage (T1a, T1b–T2, and T3–4), and ln(CLR). The expanded Cox model additionally included tumor size, N stage, lymphovascular invasion, and tumor necrosis. Both ridge-penalized Cox models included the same covariates as the expanded model. The penalty was selected by five-fold cross-validation: λ_1_SE denotes the largest penalty within one standard error of the minimum cross-validated deviance, whereas λmin denotes the penalty yielding the minimum cross-validated deviance. Conventional confidence intervals and P values were not calculated for the penalized estimates.
(DOCX)

S2 Table Assessment of the proportional-hazards assumption in the primary and expanded Cox models. Proportional-hazards tests were based on scaled Schoenfeld residuals using a Kaplan–Meier transformation of time. The primary models included age, Fuhrman grade (1–2 vs. 3–4), T stage (T1a, T1b–T2, and T3–4), and ln(CLR) (n = 414; 50 OS events and 46 CSS events). The expanded models additionally included tumor size, N stage, lymphovascular invasion, and tumor necrosis (n = 413; 49 OS events and 45 CSS events). Multi-level factors were tested jointly. DF, degrees of freedom; CLR, C-reactive protein-to-lymphocyte ratio; OS, overall survival; CSS, cancer-specific survival.
(DOCX)

S3 Table Time-specific associations between ln(CLR) and survival in Cox models with time-varying coefficients. All models included 414 patients, with 50 OS events and 46 CSS events. Models included age, Fuhrman grade, T stage, and ln(CLR), with interactions of Fuhrman grade, both T-stage indicators, and ln(CLR) with log(t/36). HRs represent a one-unit increase in ln(CLR); CIs are pointwise. The likelihood ratio P values for the ln(CLR) time interaction, conditional on the pathological time interactions, were 0.253 for OS and 0.331 for CSS. OS, overall survival; CSS, cancer-specific survival; CI, confidence interval.
(DOCX)

S4 Table Bootstrap internal validation of discrimination and calibration for the clinical model with and without CLR. The clinical model included age, Fuhrman grade, and T stage; the extended model additionally included ln(CLR). All analyses used 414 patients with 50 OS events. Discrimination estimates were based on 999 successful paired bootstrap repetitions out of 1,000 attempts. Calibration estimates were based on 997 successful repetitions for each model and time point. Absolute calibration errors were calculated over 100 equally spaced points spanning the 5th–95th percentiles of each model’s predicted survival probabilities. CLR, C-reactive protein-to-lymphocyte ratio; OS, overall survival.
(DOCX)

S1 Data Patient-level dataset underlying the analyses of preoperative CLR and survival outcomes in patients with non-metastatic clear cell renal cell carcinoma. (XLSX)

## Abbreviations

RCC: Renal cell carcinoma
ccRCC: Clear cell renal cell carcinoma
CLR: C-reactive protein-to-lymphocyte ratio
CRP: C-reactive protein
CNR: C-reactive protein-to-neutrophil ratio
CMR: C-reactive protein-to-monocyte ratio
NLR: Neutrophil-to-lymphocyte ratio
CAR: C-reactive protein-to-albumin ratio
OS: Overall survival
CSS: Cancer-specific survival
LVI: Lymphvascular invasion
AJCC: American Joint Committee on Cancer
TNM: Tumor–node–metastasis
HR: Hazard ratio
CI: Confidence interval
ROC: Receiver operating characteristic
AUC: Area under the curve
RCS: Restricted cubic spline
C-index: Concordance index
AIC: Akaike information criterion
LRT: Likelihood ratio test
IQR: Interquartile range
IL-6: Interleukin-6
CTL: Cytotoxic T lymphocyte
NK: Natural killer
LMR: Lymphocyte-to-monocyte ratio
PLR: Platelet-to-lymphocyte ratio
SII: Systemic immune-inflammation index
SIRI: Systemic inflammation response index
PFS: Progression-free survival
DFS: Disease-free survival
MFS: Metastasis-free survival.
