# Supplementary material for: Prognostic value of the preoperative C-reactive protein-to-lymphocyte ratio in patients with non-metastatic clear cell renal cell carcinoma
Source: PLoS One. 2026 Oct 30;21(10):e0362126. doi: 10.1371/journal.pone.0362126 (PMC13722192; doi:10.1371/journal.pone.0362126)
Supplement: Supplementary file 1 [file pone.0362126.s003.docx]

**S1 Table. Sensitivity analyses of the association between ln(CLR) and overall and cancer-specific survival.**

| **Model** | **OS HR (95% CI)** | ***P* value** | **CSS HR (95% CI)** | ***P* value** |
| --- | --- | --- | --- | --- |
| Primary Cox model | 1.573 (1.242–1.993) | <0.001 | 1.596 (1.251–2.036) | <0.001 |
| Expanded Cox model | 1.623 (1.240–2.122) | <0.001 | 1.638 (1.245–2.154) | <0.001 |
| Expanded ridge Cox model (λ₁SE) | 1.121 | — | 1.112 | — |
| Expanded ridge Cox model (λmin) | 1.250 | — | 1.305 | — |

All models were fitted in the same complete-case sample of 413 patients, with 49 OS events and 45 CSS events. HRs represent a one-unit increase in ln(CLR). The primary Cox model included age, Fuhrman grade (1–2 vs. 3–4), T stage (T1a, T1b–T2, and T3–4), and ln(CLR). The expanded Cox model additionally included tumor size, N stage, lymphovascular invasion, and tumor necrosis. Both ridge-penalized Cox models included the same covariates as the expanded model. The penalty was selected by five-fold cross-validation: λ₁SE denotes the largest penalty within one standard error of the minimum cross-validated deviance, whereas λmin denotes the penalty yielding the minimum cross-validated deviance. Conventional confidence intervals and P values were not calculated for the penalized estimates.
