# Supplementary material for: Prognostic value of the preoperative C-reactive protein-to-lymphocyte ratio in patients with non-metastatic clear cell renal cell carcinoma
Source: PLoS One. 2026 Oct 30;21(10):e0362126. doi: 10.1371/journal.pone.0362126 (PMC13722192; doi:10.1371/journal.pone.0362126)
Supplement: Supplementary file 2 [file pone.0362126.s004.docx]

**S2 Table. Assessment of the proportional-hazards assumption in the primary and expanded Cox models.**

| **Outcome** | **Model** | **Term** | **χ²** | **DF** | **P value** |
| --- | --- | --- | --- | --- | --- |
| OS | Primary Cox | Age | 0.653 | 1 | 0.419 |
| OS | Primary Cox | Fuhrman grade | 6.062 | 1 | 0.014 |
| OS | Primary Cox | T stage | 11.256 | 2 | 0.004 |
| OS | Primary Cox | ln(CLR) | 4.923 | 1 | 0.026 |
| OS | Primary Cox | Global test | 13.083 | 5 | 0.023 |
| CSS | Primary Cox | Age | 2.455 | 1 | 0.117 |
| CSS | Primary Cox | Fuhrman grade | 7.630 | 1 | 0.006 |
| CSS | Primary Cox | T stage | 8.719 | 2 | 0.013 |
| CSS | Primary Cox | ln(CLR) | 3.275 | 1 | 0.070 |
| CSS | Primary Cox | Global test | 12.953 | 5 | 0.024 |
| OS | Expanded Cox | Age | 0.953 | 1 | 0.329 |
| OS | Expanded Cox | Fuhrman grade | 5.898 | 1 | 0.015 |
| OS | Expanded Cox | T stage | 10.725 | 2 | 0.005 |
| OS | Expanded Cox | Tumor size | 1.774 | 1 | 0.183 |
| OS | Expanded Cox | N stage | 1.233 | 1 | 0.267 |
| OS | Expanded Cox | Lymphovascular invasion | 0.605 | 1 | 0.437 |
| OS | Expanded Cox | Tumor necrosis | 2.395 | 1 | 0.122 |
| OS | Expanded Cox | ln(CLR) | 4.864 | 1 | 0.027 |
| OS | Expanded Cox | Global test | 16.213 | 9 | 0.063 |
| CSS | Expanded Cox | Age | 2.761 | 1 | 0.097 |
| CSS | Expanded Cox | Fuhrman grade | 7.091 | 1 | 0.008 |
| CSS | Expanded Cox | T stage | 7.800 | 2 | 0.020 |
| CSS | Expanded Cox | Tumor size | 0.412 | 1 | 0.521 |
| CSS | Expanded Cox | N stage | 1.151 | 1 | 0.283 |
| CSS | Expanded Cox | Lymphovascular invasion | 0.092 | 1 | 0.762 |
| CSS | Expanded Cox | Tumor necrosis | 0.032 | 1 | 0.859 |
| CSS | Expanded Cox | ln(CLR) | 2.992 | 1 | 0.084 |
| CSS | Expanded Cox | Global test | 16.475 | 9 | 0.058 |

Proportional-hazards tests were based on scaled Schoenfeld residuals using a Kaplan–Meier transformation of time. The primary models included age, Fuhrman grade (1–2 vs. 3–4), T stage (T1a, T1b–T2, and T3–4), and ln(CLR) (n=414; 50 OS events and 46 CSS events). The expanded models additionally included tumor size, N stage, lymphovascular invasion, and tumor necrosis (n=413; 49 OS events and 45 CSS events). Multi-level factors were tested jointly. DF, degrees of freedom; CLR, C-reactive protein-to-lymphocyte ratio; OS, overall survival; CSS, cancer-specific survival.
