# Supplementary material for: Prognostic value of the preoperative C-reactive protein-to-lymphocyte ratio in patients with non-metastatic clear cell renal cell carcinoma
Source: PLoS One. 2026 Oct 30;21(10):e0362126. doi: 10.1371/journal.pone.0362126 (PMC13722192; doi:10.1371/journal.pone.0362126)
Supplement: Supplementary file 3 [file pone.0362126.s005.docx]

**S3 Table. Time-specific associations between ln(CLR) and survival in Cox models with time-varying coefficients.**

| **Outcome** | **Months after surgery** | **Number at risk** | **HR (95% CI)** |
| --- | --- | --- | --- |
| OS | 12 | 408 | 1.877 (1.307–2.697) |
| OS | 36 | 364 | 1.572 (1.219–2.028) |
| OS | 60 | 237 | 1.448 (1.047–2.000) |
| CSS | 12 | 408 | 1.876 (1.303–2.699) |
| CSS | 36 | 364 | 1.594 (1.219–2.084) |
| CSS | 60 | 237 | 1.478 (1.037–2.107) |

All models included 414 patients, with 50 OS events and 46 CSS events. Models included age, Fuhrman grade, T stage, and ln(CLR), with interactions of Fuhrman grade, both T-stage indicators, and ln(CLR) with log(t/36). HRs represent a one-unit increase in ln(CLR); CIs are pointwise. The likelihood ratio P values for the ln(CLR) time interaction, conditional on the pathological time interactions, were 0.253 for OS and 0.331 for CSS. OS, overall survival; CSS, cancer-specific survival; CI, confidence interval.
