# Supplementary material for: Prognostic value of the preoperative C-reactive protein-to-lymphocyte ratio in patients with non-metastatic clear cell renal cell carcinoma
Source: PLoS One. 2026 Oct 30;21(10):e0362126. doi: 10.1371/journal.pone.0362126 (PMC13722192; doi:10.1371/journal.pone.0362126)
Supplement: Supplementary file 4 [file pone.0362126.s006.docx]

**S4 Table. Bootstrap internal validation of discrimination and calibration for the clinical model with and without CLR.**

*Panel A. Discrimination*

| **Measure** | **Apparent** | **Mean optimism** | **Optimism-corrected** |
| --- | --- | --- | --- |
| Clinical model C-index | 0.843 | 0.011 | 0.832 |
| Clinical + CLR C-index | 0.885 | 0.012 | 0.873 |
| ΔC-index | 0.042 | 0.001 | 0.041 |

*Panel B. Calibration*

| **Model** | **Time** | **Successful repetitions** | **Mean absolute calibration error** | **90th percentile of absolute error** |
| --- | --- | --- | --- | --- |
| Clinical model | 3 years | 997 | 0.0274 | 0.0347 |
| Clinical + CLR | 3 years | 997 | 0.0245 | 0.0285 |
| Clinical model | 5 years | 997 | 0.0138 | 0.0318 |
| Clinical + CLR | 5 years | 997 | 0.0149 | 0.0307 |

The clinical model included age, Fuhrman grade, and T stage; the extended model additionally included ln(CLR). All analyses used 414 patients with 50 OS events. Discrimination estimates were based on 999 successful paired bootstrap repetitions out of 1,000 attempts. Calibration estimates were based on 997 successful repetitions for each model and time point. Absolute calibration errors were calculated over 100 equally spaced points spanning the 5th–95th percentiles of each model’s predicted survival probabilities. CLR, C-reactive protein-to-lymphocyte ratio; OS, overall survival.
